# Intrinsically photosensitive retinal ganglion cells are resistant to N-methyl-D-aspartic acid excitotoxicity

**Published:** 2012-11-29

**Authors:** SW DeParis, C Caprara, C Grimm

**Affiliations:** 1 Lab of Retinal Cell Biology, Dept Ophthalmology, University of Zurich, Switzerland; 2 Zurich Center for Integrative Human Physiology, University of Zurich, Switzerland; 3 Neuroscience Center Zurich, University of Zurich and ETH Zurich, Switzerland

## Abstract

**Purpose:**

Intrinsically photosensitive retinal ganglion cells (ipRGCs) express the photopigment melanopsin (OPN4) and are mainly responsible for non-image-forming visual tasks such as circadian photoentrainment and the pupillary light reflex. Compared to other classes of RGCs, ipRGCs are more resistant to cell death in several experimental models such as ocular hypertension, optic nerve transection, and others. Here, we tested whether ipRGCs are also resistant to N-methyl-D-aspartic acid (NMDA)-induced excitotoxicity.

**Methods:**

Mice were injected intravitreally with NMDA, and subsequent expression levels of *Opn4* and *Brn3a* mRNA were analyzed with semiquantitative real-time PCR. Cells immunopositive for BRN3A and OPN4 were quantified in retinal flat mounts of NMDA- and PBS-injected eyes. The molecular response of the retina to NMDA treatment was analyzed with real-time PCR and western blotting. Intravitreal injections of wortmannin and AG-490 were used to inhibit phosphatidylinositol 3-kinase (PI3K)/AKT and Janus kinase/signal transducers and activators of transcription (JAK/STAT) signaling, respectively.

**Results:**

In contrast to retinal *Brn3a* expression and BRN3A-containing cells, levels of *Opn4* mRNA and the number of OPN4-expressing cells were not reduced after NMDA injection. Survival of ipRGCs after NMDA injection was not strain specific, did not require the presence of photoreceptor cells, and did not depend on PI3K/AKT or JAK/STAT signaling, although both signaling pathways were activated after NMDA treatment.

**Conclusions:**

Our data support the existence of an efficient survival system for ipRGCs. This system does not depend on PI3K/AKT or JAK/STAT signaling. Identification of the responsible molecular survival mechanisms may provide clues to protect “traditional” ganglion cells in diseases such as glaucoma.

## Introduction

Intrinsically photosensitive retinal ganglion cells (ipRGCs) express the photopigment melanopsin (also known as opsin 4, or OPN4) and make up about 1%–3% of the total ganglion cell population in the mammalian retina [1]. ipRGCs are morphologically diverse with several distinct functions [2]. They are primarily responsible for non-image-forming tasks such as circadian photoentrainment and the pupillary light reflex via projection to the suprachiasmatic nucleus (SCN) and olivary pretectal nucleus, respectively [1-3]. Nevertheless, some ipRGCs project to the dorsal lateral geniculate nucleus (dLGN) and superior colliculus and may be involved in low-acuity pattern vision [2]. Interestingly, ipRGCs have been found to be resistant to cell death in various experimental models such as intraocular hypertension [4,5], optic nerve transection [5,6], and kainic acid treatment [7]. ipRGCs are also less prone to death in the DBA/2J mouse, a model for glaucoma [8], and in advanced stages of human neurodegenerative ocular diseases due to mitochondrial dysfunction [9]. It remains to be investigated whether ipRGCs also survive after N-methyl-D-aspartic acid (NMDA)-induced excitotoxicity, the main experimental approach to induce and study ganglion cell death.

NMDA is an agonist at the NMDA receptor, one of three ionotropic glutamate receptors [10]. NMDA induces degeneration of ganglion and amacrine cells in the ganglion cell layer (GCL) and inner nuclear layer (INL) of the retina [11,12], and is often used to study molecular mechanisms of ganglion cell death and neuroprotection [13]. Since NMDA injury activates not only proapoptotic but also antiapoptotic signaling [14], this model is also suitable for studying survival mechanisms. Detailed characterization of the molecular response after NMDA application may thus allow an understanding of why some cells die and some cells survive in response to a particular stimulus. This seems crucial for comprehending the mechanisms of ganglion cell death and eventually treating diseases such as glaucoma, the second leading cause of blindness worldwide [15].

The molecular basis for protecting ipRGCs has not been identified, but may involve phosphatidylinositol 3-kinase (PI3K)/AKT signaling, at least after optic nerve transection and ocular hypertension [5]. Another endogenous survival signaling pathway that may increase the resistance of ipRGCs may involve Janus kinase/signal transducer and activator of transcription (JAK/STAT) signaling, which has been shown to support the survival of various retinal cells against cell death. JAK/STAT signaling is activated in response to various inner and outer retinal insults such as photoreceptor injury [16], increased intraocular pressure [17], and NMDA excitotoxicity [18,19]. This signaling is initiated by the binding of cytokines of the interleukin-6 (IL-6) family of proteins to their respective transmembrane receptors. Within the IL-6 family, leukemia inhibitory factor (LIF) in particular has been found to be critical for survival of retinal cells under stress. Photoreceptor injury induces *Lif* expression in a subset of Müller glial cells, which controls a downstream signaling cascade culminating in the increased expression of neuroprotective factors such as fibroblast growth factor (FGF2) [16,20-22]. In addition, *Lif* expression is induced after intravitreal injection of NMDA in mice [18], and STAT3 activation is protective for retinal ganglion cells after glutamate injury in vitro and ischemia-reperfusion in vivo [19]. However, whether these pathways are involved in protecting ipRGCs is not known.

In this study, we show that ipRGCs are also resistant to cell death after intravitreal injection of NMDA in mice and present data suggesting that the PI3K/AKT and JAK/STAT pathways are not major contributing factors in the enhanced survival of ipRGCs in this model.

## Methods

### Animals

Animals were treated in accordance with the regulations of the Cantonal Veterinary Authority of Zürich and with the Association for Research in Vision and Ophthalmology Statement for the Use of Animals in Ophthalmic and Vision Research. The 129S6/SvEvTac (129S6) mice (Taconic, Hudson, NY), rd10 mice (Jackson Laboratory, Bar Harbor, ME), and CD1 mice (Charles River Laboratories, Wilmington, MA) were housed in a 12 h:12 h light-dark cycle with access to food and water ad libitum. During the light cycle, the light level was maintained at 60 lux.

### Intravitreal injections

Injections were always performed between 9 and 11 AM to control for circadian variations in gene expression levels. Mice were anesthetized with a subcutaneous injection (2 μl per gram bodyweight) of a mixture of 510 μl ketamine and 60 μl xylazine. Withdrawal response to paw pinch was tested after 10 min, and when sufficiently immobilized, the animals were prepared for surgery. A surgical needle was used to create a sling for the upper eyelid, and the cornea and conjunctiva were anesthetized locally with oxybuprocaine 0.4% eye drops (Théa Pharma, Schaffhausen, Switzerland). A small area of conjunctiva 1 mm distal to the limbus was dissected away to reveal the sclera, and a sclerostomy was made with a 30-gauge needle. A 30-gauge blunt-end needle on a 10 μl Hamilton syringe was then used to inject 1 μl of 40 mM NMDA (Sigma-Aldrich, St. Louis, MO) in 0.1 M phosphate buffered saline (PBS: 2.622 g of NaH_2_PO_4_xH_2_O; 11.5 g Na_2_HPO_4_; 8 g NaCl; 0.2 g KCl per 1000 ml H_2_O) under light microscopy visualization through the pupil. The fellow eye was injected with PBS alone. Alternately, 1 μl of a mixture of 40 mM NMDA / 1 mM wortmannin (Sigma-Aldrich) in 10% dimethyl sulfoxide (DMSO, Sigma-Aldrich) and PBS or 1 µl of a mixture of 40 mM NMDA / 10 mg/ml AG-490 (LC Laboratories, Woburn, MA) in 50% DMSO and PBS were coinjected. Here, the fellow eye was injected with 40 mM NMDA in 10% DMSO and PBS, with 1 mM wortmannin in 10% DMSO and PBS or with 10 mg/ml AG-490 in 50% DMSO and PBS, respectively. The injection needle was left in place for 20 s before being slowly withdrawn. Care was taken to avoid injury to the lens or retina. After injection, the cornea was dabbed with a cotton swab and coated with a lubricating eye gel (Lacrinorm, Bausch and Lomb, Schenkon, Switzerland). Mice recovered from anesthesia on a heating pad in dimmed light conditions with frequent monitoring and were assessed daily after injection for signs of infection.

### Morphology and quantification of retinal ganglion cells

At 6 days post injection, the eyes were enucleated and fixed overnight in 4% (w/v) paraformaldehyde in PBS. After a washing step with PBS, the eyes were dehydrated in a series of increasing ethanol concentrations, washed in xylene, and fixed in paraffin. Semithin sagittal sections (500 nm) bisecting the optic nerve were prepared and stained with hematoxylin and eosin. Sections were analyzed with light microscopy, and cell bodies in the ganglion cell layer were counted from periphery to periphery in two sections per eye and averaged. A total of three eyes (n=3) were analyzed per condition. Erythrocytes and endothelial cells were excluded from counting.

### RNA isolation and semiquantitative real time–polymerase chain reaction

Mice were sacrificed at 6 h, 24 h, 48 h, or 6 days post injection. Retinas were isolated through a corneal incision and immediately frozen in liquid nitrogen. Total RNA was extracted using an RNA isolation kit (RNeasy; Qiagen, Hilden, Germany) including a DNase treatment step. Retinas from eyes injected with AG-490 were isolated, and RNA and protein were simultaneously prepared from the same retina: Retinas were homogenized in 200 µl H_2_O by sonication (10 cycles; 0.3 s ON (30% output) and 0.7 s OFF) at 4 °C. Immediately after homogenization, 50 µl were added to 450 µl lysis/binding buffer from the High Pure RNA Isolation Kit (Roche Diagnostics, Mannheim, Germany). RNA isolation was performed using the same kit according to the manufacturer’s recommendations. 140 µl of the homogenate were added to 16 µl of 1M Tris-HCl (pH 7.6), and protein concentrations were determined using Bradford reagent and a bovine serum albumin standard. Proteins were used for western blotting as described below.

Reverse transcription was performed using oligo(dT) and M-MLV reverse transcriptase (Promega, Madison, WI). Semiquantitative real-time PCR was used to analyze gene expression of samples in duplicate or triplicate. This was performed with specific primer pairs (Table 1) spanning an exon-exon junction in the RNA of the gene in question, a polymerase ready mix (LightCycler 480 SYBR Green I Master Mix; Roche Diagnostics, Indianapolis, IN), and a thermocycler (LightCycler; Roche Diagnostics). Signals were normalized to *β-actin* (*Actb*) and relative expression was calculated with the comparative threshold cycle (ΔΔCT) method using a control sample for calibration [23].

**Table 1:** Primer pairs used for semiquantitative real time PCR

Gene	Upstream	Downstream	Product
*Actb*	CAACGGCTCCGGCATGTGC	CTCTTGCTCTGGGCCTCG	153 bp
*Brn3a*	CGCCGCTGCAGAGCAACCTCTT	TGGTACGTGGCGTCCGGCTT	130 bp
*Casp1*	GGCAGGAATTCTGGAGCTTCAA	GTCAGTCCTGGAAATGTGCC	138 bp
*Clc*	GCATCAACTCCGCAGCTTAG	CTGAACGCCATAGCCAGGTCT	443 bp
*Edn2*	AGACCTCCTCCGAAAGCTG	CTGGCTGTAGCTGGCAAAG	64 bp
*Fgf2*	TGTGTCTATCAAGGGAGTGTGTGC	ACCAACTGGAGTATTTCCGTGACCG	158 bp
*Gfap*	CCACCAAACTGGCTGATGTCTAC	TTCTCTCCAAATCCACACGAGC	240 bp
*Gnat1*	GAGGATGCTGAGAAGGATGC	TGAATGTTGAGCGTGGTCAT	209 bp
*Gnat2*	GCATCAGTGCTGAGGACAAA	CTAGGCACTCTTCGGGTGAG	192 bp
*Lif*	AATGCCACCTGTGCCATACG	CAACTTGGTCTTCTCTGTCCCG	216 bp
*Mcp-1*	GGCTCAGCCAGATGCAGTTA	CTGCTGCTGGTGATCCTCTT	108 bp
*Opn4*	CCAGCTTCACAACCAGTCCT	CAGCCTGATGTGCAGATGTC	111 bp
*Stat3*	CAAAACCCTCAAGAGCCAAGG	TCACTCACAATGCTTCTCCGC	139 bp

### Immunofluorescence on sagittal sections

Treated 129S6 wild-type mice were sacrificed 6 days following injection. Eyes were enucleated and fixed overnight in 4% (w/v) paraformaldehyde in PBS. After the cornea and lens were removed, eyecups were postfixed in 4% paraformaldehyde for an additional 2 h before being transferred to 30% sucrose in 0.1 M PBS at 4 °C for 4–12 h. The eyes were then embedded in tissue-freezing medium (Leica Microsystems Nussloch GmbH, Nussloch, Germany) and frozen in a 2-methylbutane bath cooled by liquid nitrogen. Retinal sagittal sections (12 µm) were cut, placed on slides, and incubated with a blocking solution (3% normal goat serum, 0.3% Triton X-100 in 0.1 M PBS) for 1 h at room temperature (RT). For protein detection, sections were incubated at 4 °C overnight with mouse anti-BRN3A (1:100, cat no. MAB1585, Millipore, Billerica, MA) diluted in blocking solution. After three washes with PBS, slides were incubated with a secondary antibody coupled to Cy3 for 1 h at room temperature, washed, counterstained with 4',6-diamidino-2-phenylindole (DAPI), and mounted with antifade medium (10% (v/v) Mowiol 4–88; Calbiochem, San Diego, CA), in 100 mM Tris (pH 8.5), 25% glycerol (w/v), and 0.1% 1,4-diazabicyclo (2.2.2) octane. Immunofluorescent staining was analyzed with a digitalized microscope (AxioVision; Carl Zeiss Meditec, Dublin, CA).

### Western blots

Wild-type 129S6 mice were sacrificed at 6 h or 24 h following injection and the retinas isolated and snap frozen as described above. Retinas were sonified in 0.1 M Tris/HCl (pH 8.0) and analyzed for protein content using Bradford reagent. Protein extracts were mixed with sodium dodecylsulfate sample buffer and incubated for 10 min at 75 °C. Equivalent amounts of proteins were separated with sodium dodecylsulfate–polyacrylamide gel electrophoresis and transferred to nitrocellulose membranes. Membranes were blocked in 5% milk (Bio-Rad, Hercules, CA) in TBST (10 mM Tris/HCl [pH 8.0], 150 mM NaCl, and 0.05% Tween-20) for 1 h at room temperature before being incubated overnight at 4 °C in the same 5% milk solution containing the respective primary antibody. The primary antibodies used were as follows: rabbit anti-STAT3 (1:1000, no. 9132, Cell Signaling Technology, Beverly, MA), rabbit anti-pSTAT3 (1:500, no. 9131, Cell Signaling Technology), rabbit anti-STAT1 (1:1000, no. 9172, Cell Signaling Technology), rabbit anti-pSTAT1 (1:1000, no. 9171, Cell Signaling Technology), rabbit anti-caspase 1 (1:10,000, generous gift from Peter Vandenabeele, Ghent University, Belgium), mouse anti-glial fibrillary acidic protein (1:500, no. G-3893, Sigma-Aldrich), rabbit anti-pAKT_Ser473_ (1:2500, no. 9271, Cell Signaling Technology), rabbit anti-AKT (1:2500, no. 9272, Cell Signaling Technology), and mouse anti-β-actin (1:5000, no. A5441, Sigma-Aldrich). Detection was with horseradish peroxidase–conjugated secondary antibodies, and proteins were visualized using a detection kit (Western Lightning Plus-ECL, PerkinElmer Life Sciences, Boston, MA).

### Immunofluorescence and quantification of cells in retinal flat mounts

Six days after intravitreal injection, 129S6 wild-type mice were deeply anesthetized with 200 μl ketamine / 60 μl xylazine administered intraperitoneally before being perfused with 10 ml of PBS followed by 20 ml of 4% paraformaldehyde in PBS. Eyes were enucleated, incubated for 5 min in 2% paraformaldehyde in PBS, and transferred to PBS. The eyes were cut along the ora serrata and the cornea and lens were removed. The retina was then dissected from the sclera and flattened by making four radial cuts yielding a cloverleaf shape [24]. Before immunofluorescence analysis, retinal flat mounts were incubated for 1 h (at RT) in 4% paraformaldehyde in PBS and blocked with PBS containing 3% fetal bovine serum and 0.3% Triton X-100 for 1 h (at RT). They were then incubated with the appropriate primary antibodies for 48 h: mouse anti-BRN3A (1:100, cat no. MAB1585, Millipore), rabbit anti-OPN4 (1:500, generous gift from Dr. Ignacio Provencio, Charlottesville, VA), and mouse anti-NR1 (1:500, clone N308/48, NeuroMab c/o Antibodies Inc., Davis, CA). Flat mounts were washed 3 times for 10 min each in PBS and then incubated for 2 h with the respective secondary antibody (anti-mouse-Cy2, anti-rabbit-Cy3, 1:500). They were washed again with PBS before being mounted with antifade medium (Mowiol 4-88 Reagent, Sigma-Aldrich). Immunofluorescent staining was analyzed with a digitalized microscope (AxioVision, Carl Zeiss Meditec).

Quantification of BRN3A-positive cells was performed by counting labeled cells in eight 380 by 610 μm microscopic fields per retina. Fields were located at 700 μm and 1700 μm from the optic nerve head in each retinal quadrant. The cell counts of all eight fields were averaged and extrapolated to the number of cells per mm^2^ using the measured total retina area. As there are far fewer OPN4-positive cells in the retina, these were quantified by counting the total number of labeled cells per whole retina and then converting to cells per mm^2^ as above.

### Statistical analyses

Statistical analyses were performed using Prism4 software. Statistical differences of means were calculated using one-way (if three or more experimental groups and one variable) or two-way (if two or more variables) analysis of variance (ANOVA) followed by Bonferroni post-hoc testing. A two-tailed unpaired Student *t* test was used only when only two experimental groups and one variable were present. P values less than 0.05 were considered significant.

## Results

### Ganglion cell death after intravitreal injection of N-methyl-D-aspartic acid

**Figure 1:**
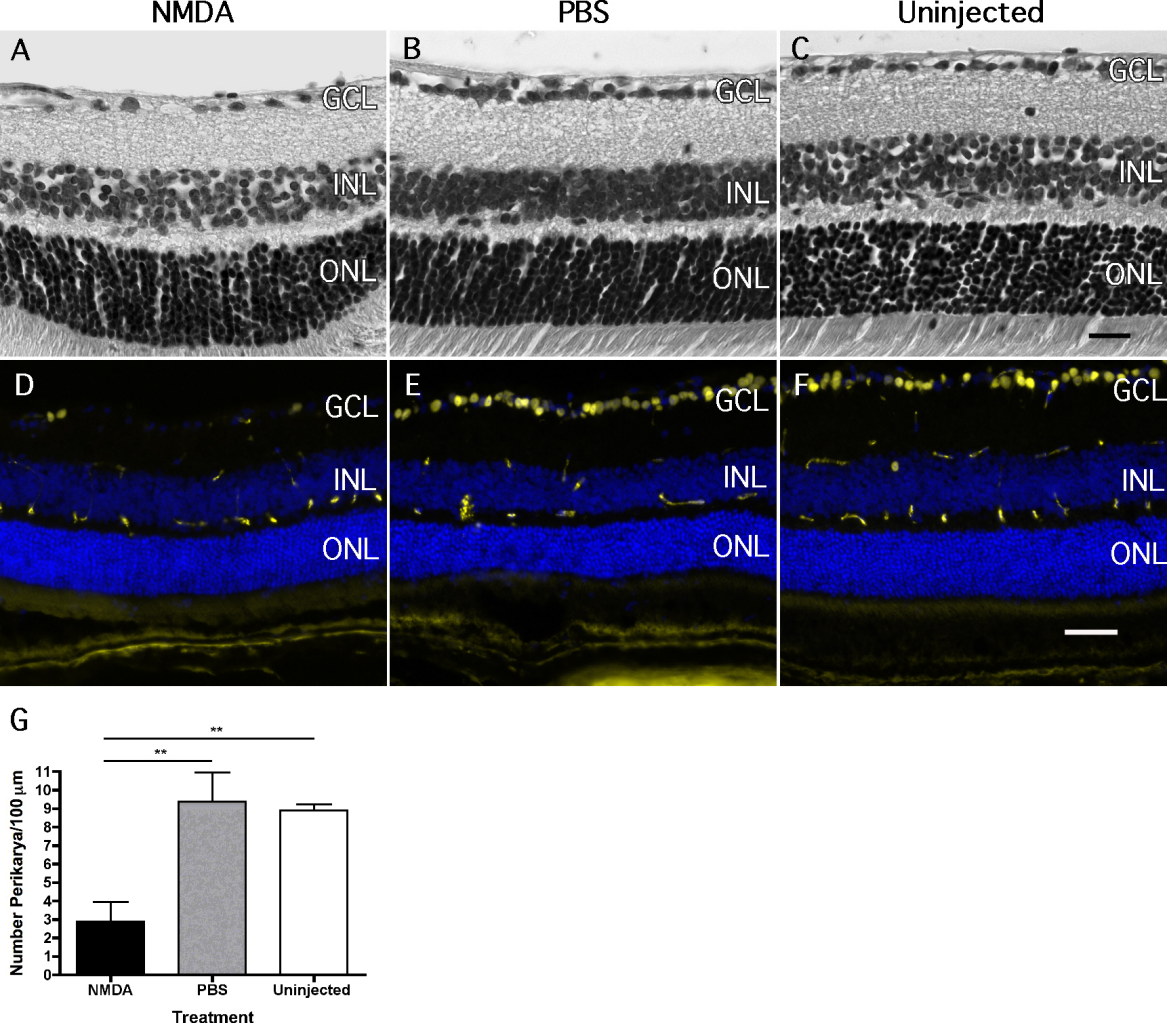
Cells in the ganglion cell layer are lost after intravitreal injection of N-methyl-D-aspartic acid. **A**-**C**: Shown are representative photomicrographs of retinal sections from wild type mice 6 days after intravitreal injection of **A**: N-methyl-D-aspartic acid (NMDA) or **B**: phosphate buffered saline (PBS). **C**: Untreated retinas served as controls. **D**-**F**: Shown are representative photomicrographs of immunfluorescent stainings for BRN3A (yellow) and DAPI (blue) in semithin sagittal sections through retinas 6 days after injection of **D**: NMDA or** E**: PBS. **F**: Uninjected eyes served as controls. Note the non-specific staining of blood vessels by the secondary antibody, especially in the outer plexiform layer (OPL), inner nuclear layer (INL), and inner plexiform layer (IPL). **G**: Cell bodies in the ganglion cell layer (GCL) were quantified at 6 days after intravitreal injection of NMDA (black bar) or PBS (grey bar), and in untreated eyes (white bar). Shown are means±SD (n=3) for all treatments and analyses. Scale bars were **A**-**C**: 20 µm, **D**-**F**: 50 µm. **: p<0.01. A one-way ANOVA with Bonferroni post hoc test was used to test statistical significance.

We confirmed loss of cells in the ganglion cell layer with light microscopy of sagittal retinal sections at 6 days after intravitreal injection of NMDA (Figure 1A-C), and with immunofluorescence staining for BRN3A (Figure 1D-F). BRN3A is a POU-domain transcription factor expressed in thalamocortical and collicular projecting RGCs. BRN3A is frequently used as an RGC marker, as a decrease in *Brn3a* mRNA levels correlates with loss of ganglion cells [25-27]. NMDA-treated retinas showed reduced cell density in the GCL (Figure 1G) and probably the INL (not quantified). No difference was observed between PBS-treated and uninjected retinas; they appeared essentially normal. As in previously published studies [11,28-30], we observed a loss of about two-thirds of cells in the ganglion cell layer after NMDA was injected compared to PBS (p<0.001). As already shown by others, this effect was dose dependent [11] (data not shown). Although we did not differentiate between ganglion cells and displaced amacrine cells in the ganglion cell layer, NMDA treatment leads to significant loss of both types of cells in the inner retina, and a loss of cells in the ganglion cell layer strongly correlates with axonal loss in the optic nerve [18].

### Expression of *Opn4* is not affected by N-methyl-D-aspartic acid injection

**Figure 2:**
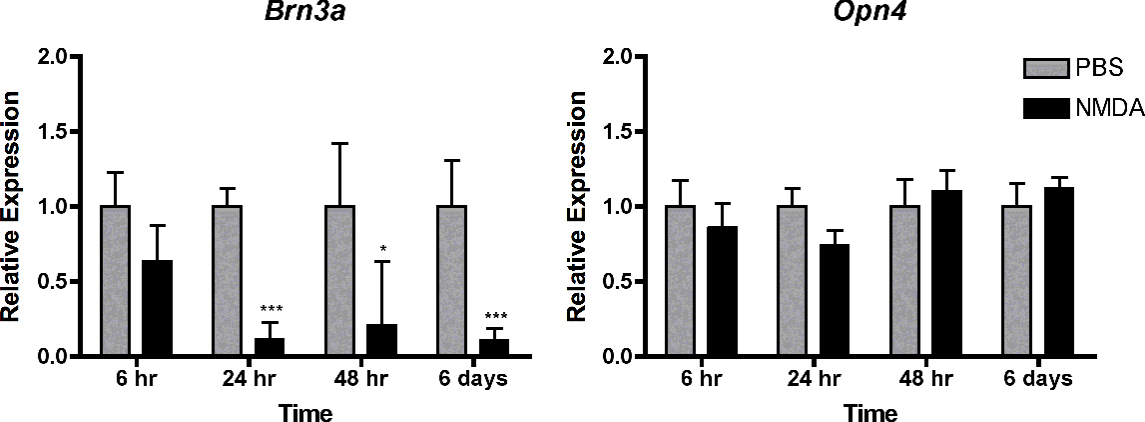
Expression of *Opn4* is not affected by N-methyl-D-aspartic acid injection. Shown are the relative expression levels of *Brn3a* and *Opn4* in retinas of wild type mice at 6 h, 24 h, 48 h, and 6 days after injection of N-methyl-D-aspartic acid (NMDA, black bars) or phosphate buffered saline (PBS, grey bars). Expression after NMDA injection was expressed relative to expression after PBS injection, which was set to 1 for each time point. Shown are means±SD of n=4-6. *; p<0.05; ***; p<0.001. A two-way ANOVA with Bonferroni post hoc test was used to test statistical significance.

To test the sensitivity of the melanopsin-expressing subset of ganglion cells to NMDA toxicity, we analyzed expression of *Brn3a* and *Opn4* mRNA via semiquantitative real-time PCR in wild-type mice at 6 h, 24 h, 48 h, and 6 days after intravitreal injection of NMDA (Figure 2). As expected, expression of *Brn3a* was strongly reduced starting at 24 h after treatment. Although apoptosis starts as early as 6 h after NMDA injection [28], the decrease in *Brn3a* mRNA expression at this early time point was not yet statistically significant. In contrast to *Brn3a*, levels of *Opn4* mRNA were unchanged at all four time points after NMDA injection, suggesting either that *Opn4*-expressing RGCs were resistant against NMDA toxicity or that the surviving cells increased expression as a compensatory reaction. Since *Opn4* is expressed in a circadian pattern [31,32], NMDA-treated and control mice of a particular time group were always treated in parallel and analyzed at the same time of day.

### OPN4-positive ganglion cells are resistant to N-methyl-D-aspartic acid–induced excitotoxic cell death

**Figure 3:**
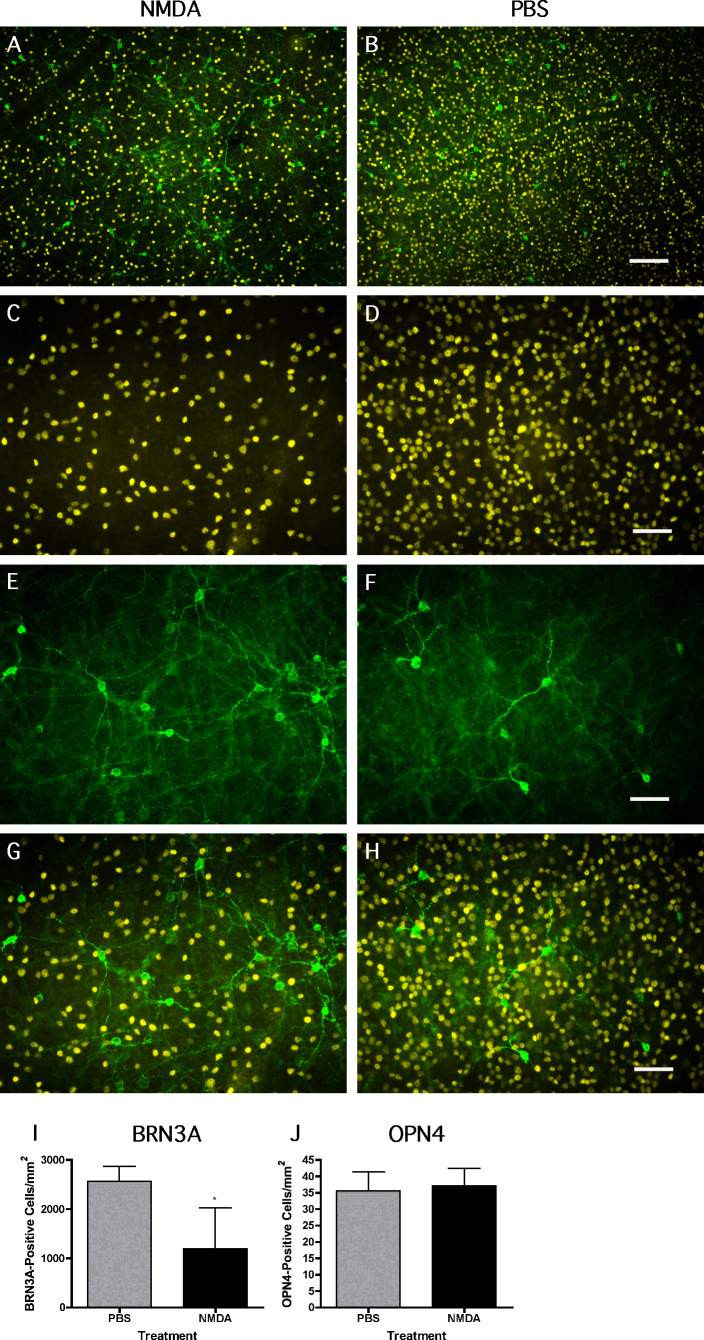
N-methyl-D-aspartic acid treatment does not reduce survival of OPN4-positive ganglion cells. Shown are representative photomicrographs taken from retinal flat mounts stained for BRNA3A (yellow) and OPN4 (green) at 6 days after **A**, **C**, **E**, **G**: N-methyl-D-aspartic acid (NMDA) injection or **B**, **D**, **F**, **H**: phosphate buffered saline (PBS) injection. **A**, **B**: Shown is double labeling for BRN3A and OPN4. **C**, **D**: Shown is BRN3A staining.** E**, **F**: Shown is OPN4 staining. **G**, **H**: Shown are merges of panels **C** and** E**, or** D** and **F**, respectively, n=3-4. Scale bars are **A**, **B**: 100 µm; **C**-**H**: 50 µm. **I**-**J**: Shown is quantification of I: BRN3A- positive cells and **J**: OPN4-positive cells in retinal flat mounts at 6 days after NMDA (black bars) or PBS (grey bars) treatment. Shown are means±SD of n=3-4. *; p<0.05. An unpaired two-tailed Student t test was used to test statistical significance.

To distinguish between resistance against NMDA toxicity and a compensatory upregulation of *Opn4* in surviving RGCs, we costained flat mounted retinas of NMDA- and PBS-injected mice for BRN3A and OPN4 (Figure 3A-H). We observed markedly fewer BRN3A-positive cells in NMDA-treated retinas (Figure 3A,C,G) compared to the control retinas (Figure 3B,D,H), but no obvious difference in the number of OPN4-positive cells between the two treatment groups (Figure 3A,E,G,B,F,H). Quantification of BRN3A- and OPN4-positive cells confirmed the mRNA expression data, showing a significantly reduced number of BRN3A-positive cells in the retinas of the NMDA-treated mice (1188±834.6 cells/mm^2^ for NMDA versus 2567±306 cells/mm^2^ for PBS; mean±SD; Figure 3I) while the number of OPN4-positive cells did not change (37.1±2.3 cells/mm^2^ for NMDA versus 35.5±3.3 cells/mm^2^ for PBS; Figure 3J). Thus, *Opn4* RNA levels were maintained after NMDA treatment not because of a compensatory upregulation of gene expression but because of the resistance of OPN4-positive ipRGCs to NMDA excitotoxicity.

### Intrinsically photosensitive retinal ganglion cell resistance to N-methyl-D-aspartic acid toxicity is independent of genetic background, pigmentation, and the presence of photoreceptor cells

**Figure 4:**
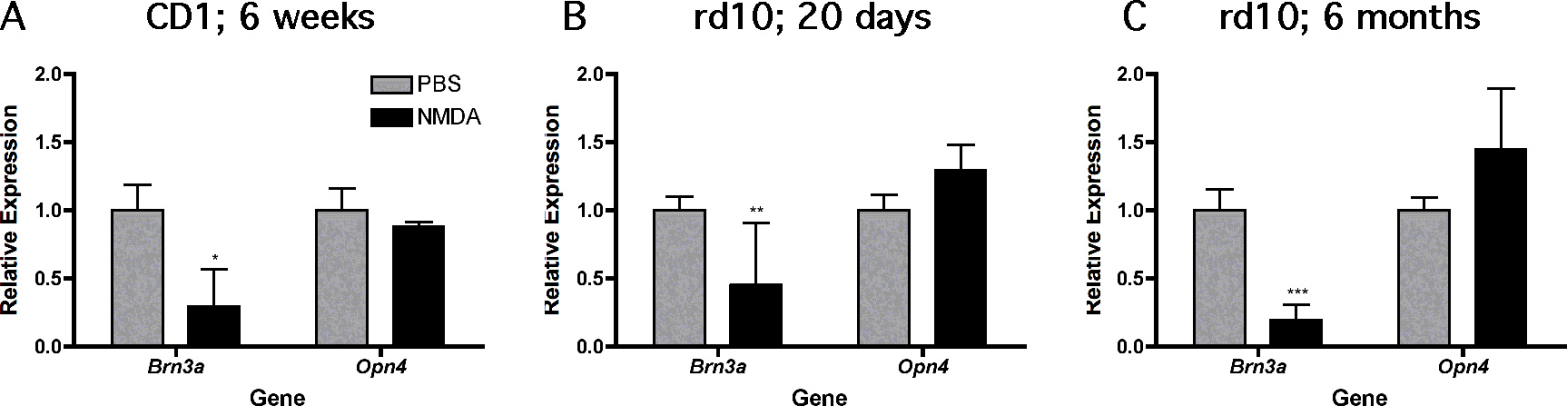
Resistance of OPN4-positive cells against N-methyl-D-aspartic acid toxicity does not depend on genetic background, pigmentation or presence of photoreceptor cells. **A**: Shown is the relative expression of *Brn3a *and *Opn4* mRNAs in retinas of 6 week old albinotic CD1 wild type mice at 6 days after injection of N-methyl-D-aspartic acid (NMDA, black bars) or phosphate buffered saline (PBS, grey bars). **B**: Shown is the relative expression of *Brn3a* and *Opn4* at 6 days after injection of NMDA (black bars) or PBS (grey bars) in rd10 mice at 20 days of age.** C**: Shown is the relative expression of *Brn3a* and *Opn4* at 6 days after injection of NMDA (black bars) or PBS (grey bars) in rd10 mice at 6 months of age. Gene expression after NMDA injection was expressed relative to expression after PBS injection, which was set to 1. Shown are means±SD of n=4-10. *: p<0.05; **: p<0.01; ***: p<0.001. Statistical tests used were** A**: unpaired two-tailed Student t test, and **B**-**C**: two-way ANOVA with Bonferroni post hoc test.

To determine whether the survival of OPN4-positive ipRGCs after NMDA treatment was a phenomenon isolated to the particular strain of wild-type mice used (129S6), we also analyzed *Brn3a* and *Opn4* expression in NMDA-treated albino CD1 mice. Again, NMDA treatment significantly reduced *Brn3a* but not *Opn4* expression (Figure 4A). This observation suggests that the survival of ipRGCs after NMDA is a general phenomenon and is not due to differences in pigmentation or genetic background.

To determine whether ipRGC resistance to NMDA toxicity depended on the presence of regulated glutamate release from bipolar cells and thus on phototransduction-initiated signaling from photoreceptor cells, we injected NMDA in rd10 mice. The rd10 mouse carries a missense mutation in exon 13 of the β-subunit of cyclic guanosine monophosphate phosphodiesterase, and exhibits degeneration of rod and cone photoreceptors beginning at PND16 with almost complete degeneration by PND60 [33]. We injected the eyes of rd10 mice at PND20 when the retinas contained functional photoreceptors and at 6 months of age, when the rd10 mice had virtually no photoreceptor function left. We confirmed degeneration of photoreceptors with semiquantitative real-time PCR for rod (*Gnat1*) and cone (*Gnat2*) transducin, which were expressed at the expected levels [34] in each age group (data not shown). Six days after injection, we compared *Brn3a* and *Opn4* expression in young and old rd10 mice. As in the wild-type mice, we observed a significant reduction in *Brn3a* but not *Opn4* mRNA expression in both age groups (Figure 4B,C). This suggests that neither NMDA-induced toxicity to “traditional” ganglion cells nor survival of ipRGCs depended on signaling from photoreceptor cells.

### Endogenous rescue and stress pathways are activated after intravitreal N-methyl-D-aspartic acid injection

**Figure 5:**
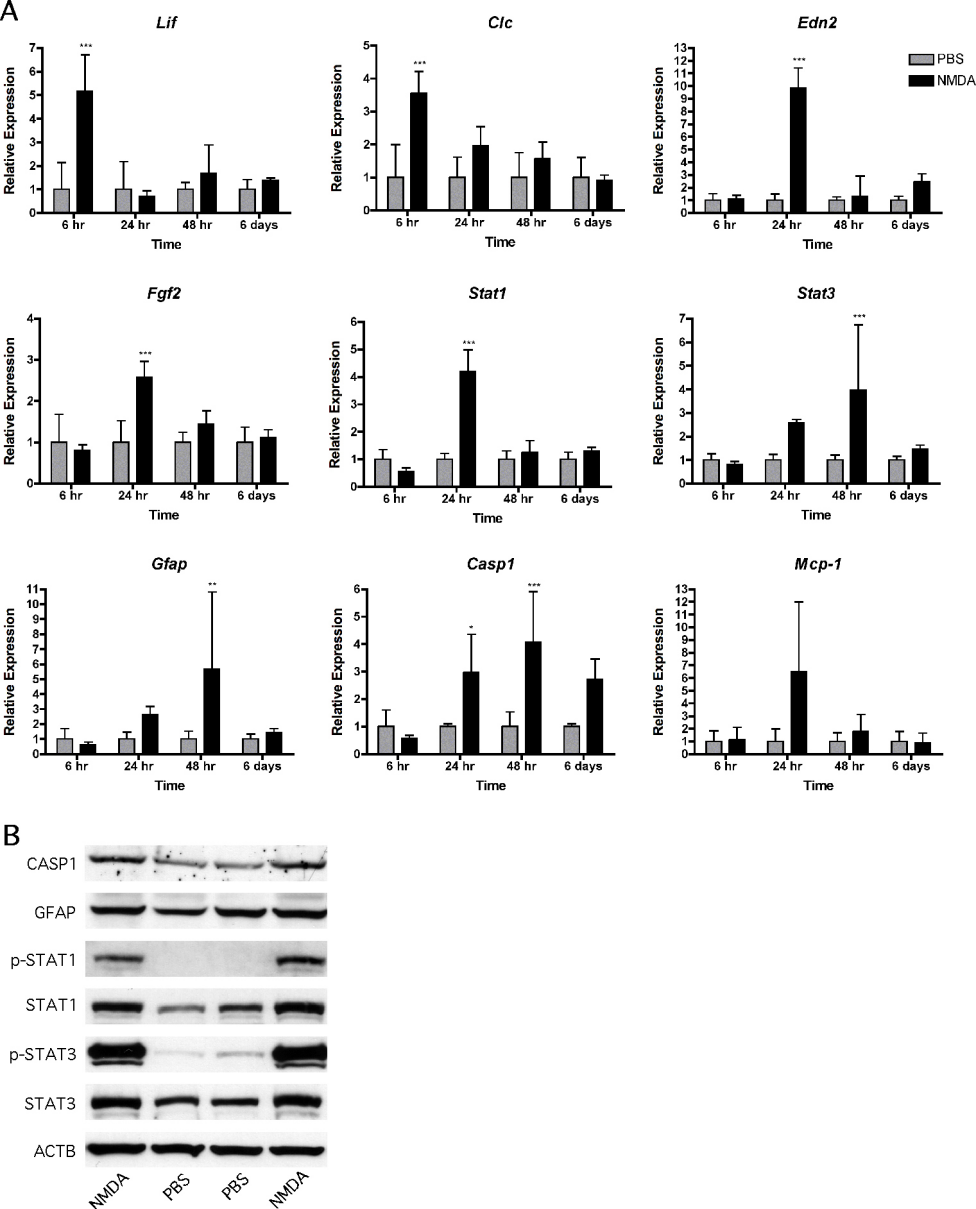
Intravitreal N-methyl-D-aspartic acid injection activates endogenous rescue and stress pathways.** A**: Shown is the relative expression *of Lif, Clc, Edn2, Fgf2, Stat1, Stat3, Gfap, Casp1,* and *Mcp-1* in retinas of 129S6 wild type mice at 6 h, 24 h, 48 h, and 6 days after injection of N-methyl-D-aspartic acid (NMDA, black bars) or phosphate buffered saline (PBS, grey bars). Expression after NMDA injection was expressed relative to expression after PBS injection, which was set to 1 for each time point. Shown are means±SD of n=4-6. *: p<0.05; **: p<0.01; ***: p<0.001. Two-way ANOVA with Bonferroni post hoc test was used to test statistical significance. **B**: Levels of proteins and phosphoproteins in total retinal extracts from 129S6 wild type mice were tested by Western Blotting at 24 h after injection of NMDA or PBS. Shown are protein levels in extracts of two retinas after NMDA and two retinas after PBS injection as indicated, n=3.

The JAK/STAT pathway is an endogenous survival signaling pathway activated in response to various inner and outer retinal insults such as photoreceptor injury [16,35] and ganglion cell death after intraocular hypertension [17]. To test a potential role of this signaling mechanism in NMDA-induced excitotoxicity, we analyzed the mRNA levels of several members of the JAK/STAT pathway at various time points after intravitreal NMDA injection (Figure 5A). We found that the *Lif* and *Clc* mRNA levels were significantly increased by a factor of 5 and 3.5, respectively, at 6 h after injection. *Edn2* and *Fgf2* mRNA expression peaked at 24 h, with approximately ten- and threefold greater expression levels compared to the PBS-injected retinas. This was followed by an increase in *Stat3* and *Gfap* expression, which peaked at 48 h. STAT3 is known to have antiapoptotic effects via activation of the suppressor of cytokine signaling (SOCS) family of proteins and the Bcl-2 family [19]. Glial fibrillary acidic protein (*Gfap*) is a marker for activated Müller glial cells. Several genes encoding proapoptotic proteins also increased expression after NMDA injection: Levels of *Stat1* mRNA were significantly increased at 24 h, and caspase-1 (*Casp-1*) mRNA was threefold and fourfold elevated compared to controls at 24 h and 48 h, respectively. In contrast, monocyte chemotactic protein-1 (*Mcp-1*), a cytokine involved in recruiting white blood cells to sites of infection or inflammation [36], was similarly expressed in the NMDA- and PBS-treated retinas, even though a tendency for increased expression was detected in NMDA retinas at 24 h after injection.

Activation of some of these molecules after NMDA injection was also detectable at the protein level with western blotting (Figure 5B). At 24 h after injection, we found strongly elevated levels of phospho-STAT3, STAT3, phospho-STAT1, and STAT1 in the NMDA-treated retinas compared to the PBS-injected controls. In addition, expression of glial fibrillary acidic protein and the proform of CASP1 was also increased, although somewhat less robustly than the proteins mentioned above.

### Intrinsically photosensitive retinal ganglion cell survival after N-methyl-D-aspartic acid injection is independent of phosphatidylinositol 3-kinase/AKT or STAT3 signaling

**Figure 6:**
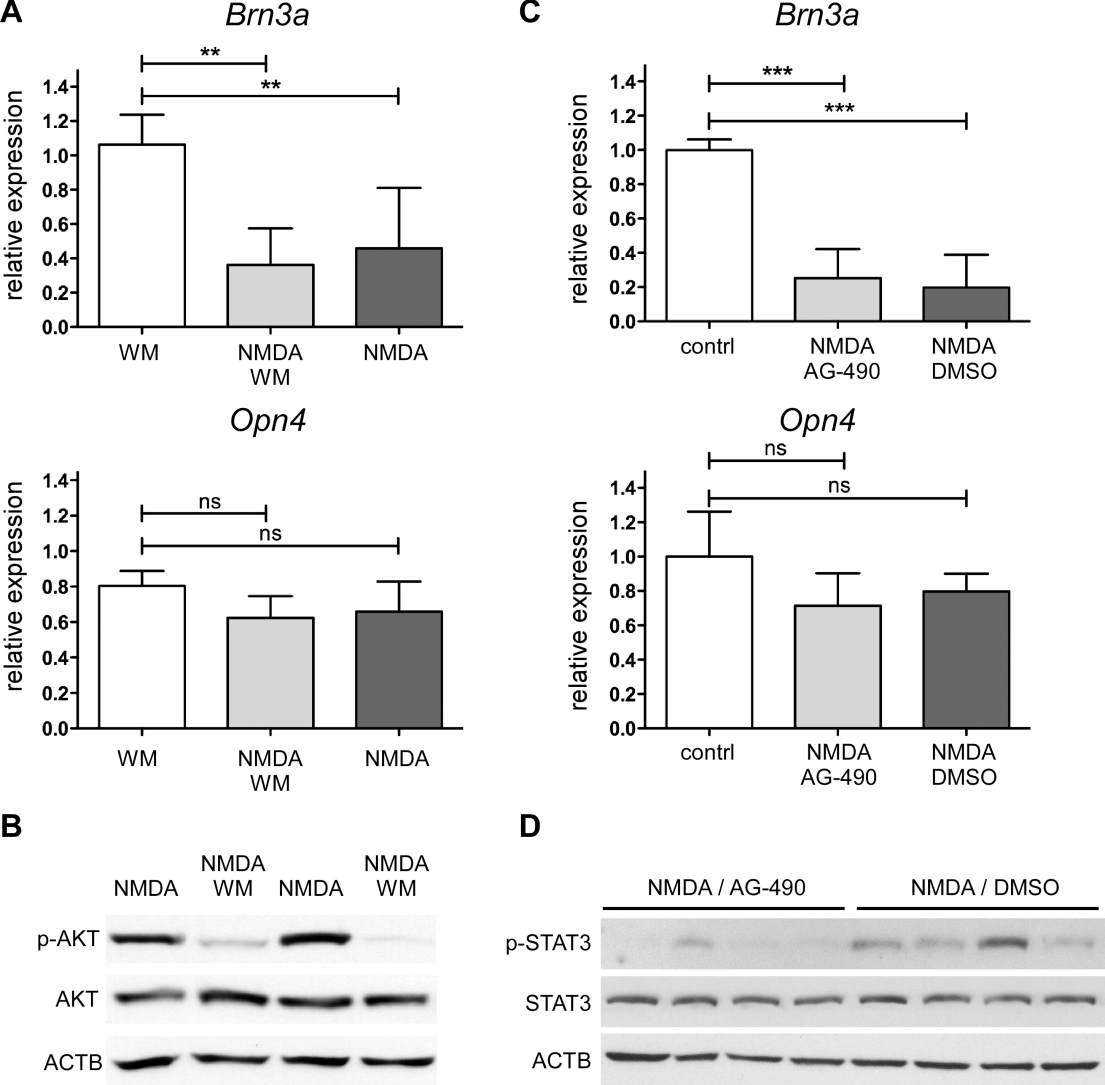
Survival of OPN4-expressing intrinsically photosensitive retinal ganglion cells does not depend on phosphatidylinositol 3-kinase/AKT and Janus kinase/signal transducer and activator of transcription signaling. **A**: Shown is the relative expression of *Brn3a* and *Opn4* in retinas of 129S6 wild type mice at 24 h after injection of N-methyl-D-aspartic acid (NMDA, black bars), NMDA plus wortmannin (WM, grey bars), or WM (white bars). Shown are means±SD of n=4-8. **: p<0.01. One-way ANOVA with Bonferroni post hoc test was used to test statistical significance. **B**: Shown are levels of proteins and phosphoproteins in total retinal extracts from 129S6 wild type mice at 6 h after injection of NMDA or NMDA plus WM as indicated, n=3. **C**: Shown is the relative expression of *Brn3a* and *Opn4 *in retinas of 129S6 wild type mice at 48 h after injection of NMDA in 50% DMSO (black bars), NMDA plus AG-490 in 50% DMSO (grey bars), or non-injected controls (white bars). Shown are means±SD of n=4. ***: p<0.001. One-way ANOVA with Bonferroni post hoc test was used to test statistical significance. **D**: Shown are levels of STAT3 and pSTAT3 in total retinal extracts from 129S6 wild type mice at 48 h after injection of NMDA or NMDA plus AG-490 as indicated. **C**: RNA and** D**: proteins were simultaneously isolated from the same retinas for analysis, n=4.

In models of optic nerve transection and ocular hypertension, the PI3K/AKT pathway was implicated in enhanced survival of ipRGCs [5]. To test whether this pathway may also contribute to the resistance of ipRGCs against NMDA toxicity, we coinjected NMDA with wortmannin (WM), an inhibitor of PI3Ks, and compared the mRNA levels of *Brn3a* and *Opn4* to retinas treated with NMDA or WM alone (Figure 6A). Although *Brn3a* levels were decreased with NMDA and NMDA plus WM injections as expected, *Opn4* remained at control levels even in the presence of the inhibitor. To confirm the inhibitory action of WM on AKT activation, we tested levels of p-AKT_Ser473_ with western blotting. At 6 h after injection, p-AKT_Ser473_ levels were high in NMDA (as previously established [14]), but not in NMDA plus WM injected retinas, indicating that the inhibitor did indeed function as expected (Figure 6B).

Injection of NMDA activated JAK/STAT signaling in the retina (Figure 5), and expression of a constitutively active form of STAT3 protected retinal ganglion cells against ischemia reperfusion in vivo and glutamate toxicity in vitro [19]. We coinjected eyes with NMDA and AG-490, an inhibitor of JAK2, to test whether activation of the JAK/STAT pathway is essential for ipRGC survival in vivo. Coinjection of NMDA with AG-490 reduced phosphorylation of STAT3 compared to injection of NMDA alone suggesting that AG-490 inhibited JAK2 signaling (Figure 6D). However, inhibition of JAK2 did not influence expression of *Brn3a* and *Opn4* after NMDA injection as indicated by the respective RNA levels at 48 h after injection (Figure 6C).

In summary, these results suggest that PI3K/AKT and STAT3 signaling may not be crucial factors in the survival of ipRGCs after NMDA injection.

**Figure 7:**
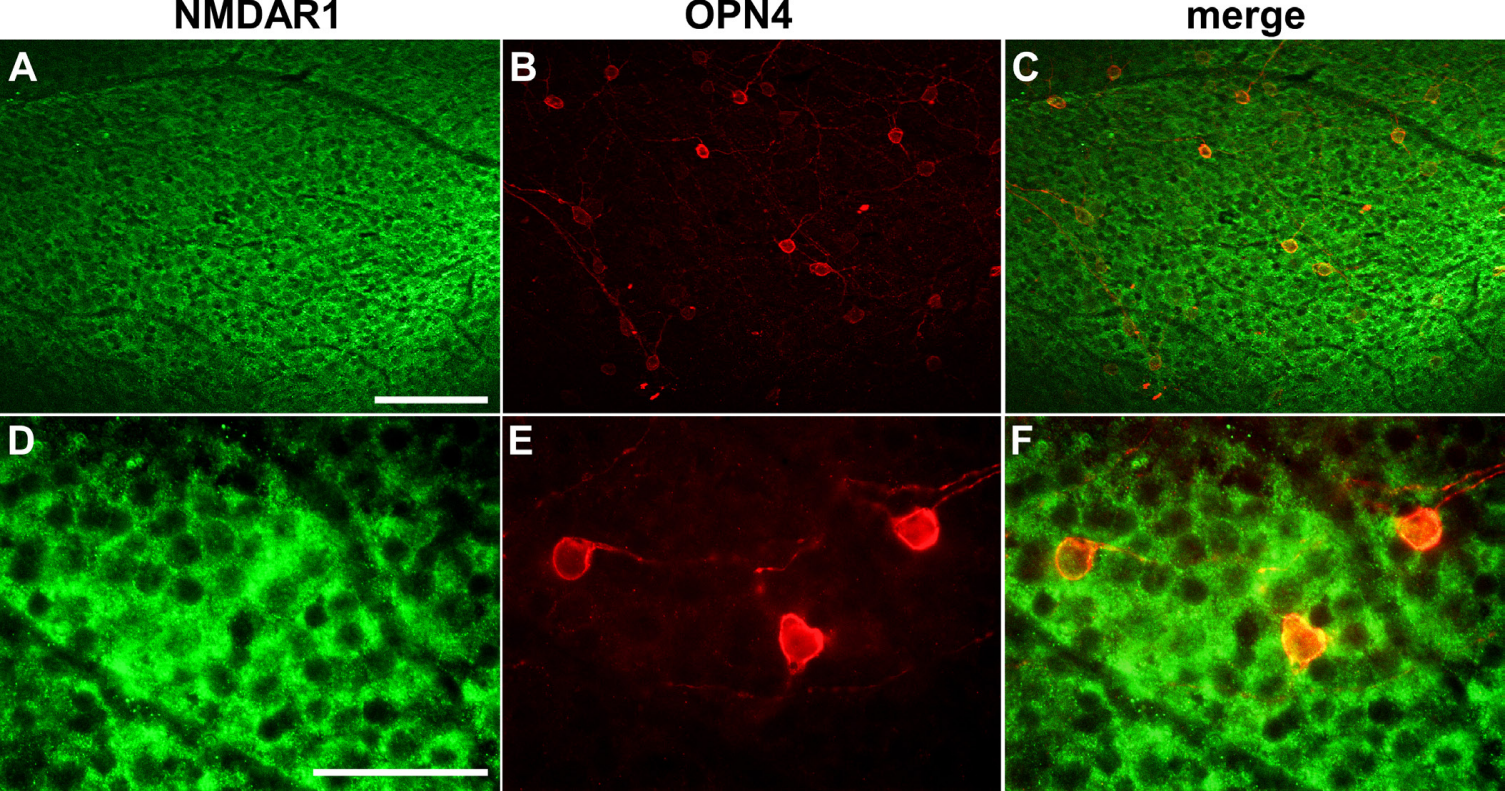
NMDAR1 colocalizes with OPN4 in retinal ganglion cells. Shown are representative photomicrographs taken from retinal flat mounts of untreated wild type mice stained for **A**, **D**: NMDAR1 (green) and **B**, **E**: OPN4 (red) at **A**-**C**: lower and **D**-**F**: higher magnification. **C**, **F**: Images shown in panels **A** and **B**, and in panels **D** and **E**, respectively, are merged. Focal plane was at the ganglion cell layer. Scale bars are **A**-**C**: 100 µm and **D**-**F**: 50 µm.

To verify expression of NMDA receptors on ipRGCs, we treated retinal flat mounts of wild-type mice with anti-NMDAR1 and anti-OPN4 antibodies and analyzed the resulting staining in the GCL. NMDAR1 was widely expressed in cell bodies but not the nuclei of the cells in the GCL (Figure 7A,D). As shown before (Figure 3), OPN4-positive cells were rare but easily detectable (Figure 7B,E). Merged images suggest that OPN4-positive cells also express NMDAR1 subunits (Figure 7C,F).

## Discussion

A growing body of evidence suggests that ipRGCs have a generally increased survival rate in various experimental models of ganglion cell death [4-8,37], as well as in human mitochondrial optic neuropathies [9]. Here we demonstrate that ipRGCs are also resistant to NMDA-induced excitotoxicity, and that their resistance does not depend on PI3K/AKT or JAK/STAT signaling. The survival of ipRGCs after various insults is intriguing, and identifying the molecular mechanism(s) responsible for their protection might provide the knowledge necessary to preserve ganglion cells in human diseases such as glaucoma.

The mechanism for NMDA excitotoxicity involves activating the NMDA receptor, which results in an influx of calcium into the cell, triggering various signaling cascades resulting in apoptotic cell death [30,38,39]. Lack of the NMDA receptor might therefore be a possible explanation for the resistance of ipRGCs to NMDA toxicity. However, several studies including this one (Figure 7) have shown that ipRGCs express glutamate receptors, and single cell PCR data specifically indicates expression of NMDA receptors by ipRGCs [3,40,41]. This suggests that the observed resistance of ipRGCs is based on another mechanism. The calcium permeability of NMDA receptors is reduced when the tripartite receptor complex interacts with NR3A, a subtype of the NR3 component of the receptor [10,42]. Accordingly, lack of NR3A increased susceptibility of RGCs to NMDA toxicity at lower NMDA concentrations of up to 2 nmol [42]. Although the effect was lost at higher NMDA levels [42], it would be interesting to analyze ipRGC survival in NR3A knockouts, especially since Jakobs and coworkers reported expression of NR3A by ipRGCs [41]. Whether increased expression of NR3A and/or reduced expression of other NMDA receptor subunits contributes to protecting ipRGCs against NMDA toxicity must be conclusively shown. However, since such a mechanism may not explain the increased resistance of ipRGCs across the many other models of ganglion cell death (see above), it seems more likely that ipRGCs have developed other mechanisms for their protection against degeneration.

Several such mechanisms have been suggested to explain the greater robustness of ipRGCs. Li and coworkers, for example, implicated the PI3K/AKT pathway in ipRGC survival after optic nerve transection and in a model of intraocular hypertension [5]. However, injection of wortmannin strongly reduced AKT phosphorylation after NMDA application but did not reduce survival of ipRGCs (Figure 6) suggesting that AKT signaling is not the main component of ipRGC resistance against NMDA toxicity.

Other published data point to an involvement of pituitary adenylate cyclase activating polypeptide (PACAP), a peptide found specifically in ipRGCs of the retinohypothalamic tract [37,43]. Exogenous administration of PACAP has been shown to be neuroprotective for “traditional” ganglion cells after optic nerve transection [44], intraocular hypertension [45], kainic acid treatment [46], and NMDA application [28]. Interestingly, exogenous administration of PACAP stimulates IL-6 production by Müller cells in the retina in vitro and in vivo [47]. IL-6 is a known activator of the JAK/STAT pathway, which may confer protection for photoreceptors and ganglion cells [16,17,21]. Several members of this endogenous rescue pathway were activated in response to NMDA injection. As reported by others [19], we observed strongly increased phosphorylation of STAT3 after NMDA application. In addition, *Lif* was expressed very early, followed by *Edn2* and *Fgf2* (Figure 5), which is similar to models of photoreceptor injury [16,35]. Thus, a signaling mechanism involving Müller glial cells may be activated not only by photoreceptor degeneration but also after NMDA injection. However, blocking JAK/STAT signaling by the application of AG-490 did not reduce survival of ipRGCs after NMDA treatment. Since we also observed elevated levels of proapoptotic proteins such as pSTAT1 and CASP1, NMDA administration activated pro- and antiapoptotic signaling [14]. The nature of the cells that activated the individual signaling pathways still need to be determined in future experiments.

Although RGCs and ipRGCs receive signaling input from rods and cones via synaptic contacts with bipolar and amacrine cells [1,48], survival of ganglion cells is mostly not affected in models of photoreceptor degeneration. However, some species differences seem to exist regarding *Opn4* expression in the absence of photoreceptors. Studies in RCS rats suggest reduced *Opn4* levels despite constant numbers of ipRGCs in the degenerated retina [32]. In addition, N-methyl-N-nitrosourea (MNU) treatment reduced expression of *Opn4* by 83%, whereas only about one-third of melanopsin-expressing cells were lost after MNU injection [49]. Although MNU primarily induces degeneration of photoreceptors, whether this loss of ipRGCs was a direct consequence of MNU or was indirectly caused by photoreceptor degeneration remains to be shown. In contrast, retinas of rod- and cone-less [50,51] as well as of rd10 mice (data not shown) show expression of *Opn4* similar to wild-type mice. Thus, ipRGCs in mice may not be directly influenced by phototransduction-related signaling from photoreceptors and/or regulated glutamate release from second-order neurons. Our data from old rd10 mice support this conclusion and show that survival of ipRGCs after NMDA treatment does not depend on normal retinal physiology and photoreceptor function.

In conclusion, ipRGCs are functionally and morphologically different from “traditional” ganglion cells in that ipRGCs survive high concentrations of intravitreal NMDA. This survival does not depend on PI3K/AKT or JAK/STAT signaling. Clearly, ipRGCs have an intrinsic strength to survive various insults toxic to “traditional” RGCs. Identifying the mechanisms conferring this increased survival competence may prove highly valuable to define strategies for protecting ganglion cells by exogenous approaches.
